# Pathological Findings and Management of COVID-19 Patients: A Brief Overview of Modern-day Pandemic

**DOI:** 10.7759/cureus.8136

**Published:** 2020-05-15

**Authors:** Muhammad S Mian, Laiba Razaq, Safeera Khan, Nadia Hussain, Mahrukh Razaq

**Affiliations:** 1 Miscellaneous, Akhtar Saeed Medical and Dental College, Lahore, PAK; 2 Internal Medicine, Akhtar Saeed Medical and Dental College, Gujranwala, PAK; 3 Internal Medicine, California Institute of Behavioral Neurosciences and Psychology, Fairfield, USA; 4 Family Medicine, Wapda Hospital, Gujranwala, PAK; 5 Obstetrics and Gynecology, Tehsil Headquarter Hospital, Gujranwala, PAK

**Keywords:** coronavirus, covid-19, ards, viral pneumonia, hydroxychloroquine, pandemic, corona pandemic, passive immunization

## Abstract

Today the world is facing one of the deadliest pandemics caused by COVID-19. This highly transmissible virus has an incubation period of 2 to 14 days. It acts by attaching to the angiotensin-converting enzyme (ACE2) with the help of glycoprotein spikes, which it uses as a receptor. Real-time polymerase chain reaction (PCR; rt-PCR) is the gold standard diagnostic test, and chest X-ray and computed tomography (CT) scan are the other main investigations. Several medications and passive immunization are in use to treat the condition.

We searched using PubMed and Google Scholar using keywords such as COVID-19, coronavirus, and their combination with pathological findings, clinical features, management, and treatment to search for relevant published literature. After the removal of duplications and the selection of only published English literature from the past five years, we had a total of 31 papers to review. Most of the COVID-19 affected patients have mild pneumonia symptoms, and those with severe disease have comorbidities. Patients with COVID-19 had pathological findings, like ground-glass opacities, consolidations, pleural effusion, lymphadenopathy, and interstitial infiltration of inflammatory cells. Radiological changes show lung changes such as consolidations and opacities, and the pathological findings were infiltration of inflammatory cells and hyalinization. Patients with mild symptoms should self-quarantine, whereas those with severe acute respiratory distress syndrome (ARDS) are treated in the hospital. Medications under trial include antivirals, antibacterials, antimalarials, and passive immunization. Supportive treatment such as oxygen therapy, extracorporeal membrane oxygenation, and ventilator support can also be used. The symptoms shown by patients are very mild and self-limiting. There is no definitive treatment, although a combination of hydroxychloroquine and azithromycin have shown good results, and passive immunization also shows promising results, their safety profile is yet to be studied in detail.

## Introduction and background

As of April 16 2020, there are a total of 1,991,562 confirmed cases and 130,885 reported deaths due to this deadly virus, which is given the name of COVID-19 [1]. The fatality rate of the virus is 2.3% [2]. The death rate is higher in people of age above 80 and those having comorbid conditions like hypertension, diabetes, and chronic respiratory disorders [2]. Approximately 48% of the people who have had fatal outcomes had a comorbid condition, 30% were hypertensive, 19% were diabetics, and 8% had coronary heart disease [3].

This virus belongs to the family of viruses called Coronaviridae. There are four genera of coronaviruses (ɑ,𝛃,ɣ,𝝳), COVID-19 belongs to the beta-coronaviruses as its genome matches 88% and 50% to the other coronaviruses which are SARS and MERS, respectively [4]. It is believed that initially, the virus transmitted from animals to humans, and among humans, the virus transmitted via respiratory droplets [3,5].

The virus has very high transmissibility as it has affected the whole world in a brief period. When infected, there is an incubation period of 2-14 days after which the patient develops the symptoms of high fever, cough, shortness of breath, myalgias, and fatigue, and the virus is contagious during the incubation period [6]. The COVID-19 in susceptible patients causes pneumonia, which rapidly progresses to acute respiratory distress syndrome (ARDS). 

The exact pathogenesis is currently unknown; however, it is suggested that it is most likely to be similar to that of SARS CoV and MERS CoV, which attach to ACE 2 enzyme on the cell surface with the help of their glycoprotein spikes. After the entry of the virus, its antigens are presented to major histocompatibility complex (MHC) via the antigen-presenting cells (APC). Then these antigens are recognized by specific cytotoxic lymphocytes that destroy the infected cells [4]. On chest X-ray and chest computed tomography (CT), ground-glass opacities, patchy consolidation, and interstitial changes at the periphery of the lungs are typical findings [7]. Real-time polymerase chain reaction (RT-PCR) is used to make the diagnosis of the suspected cases [4]. 

There is currently no recommended treatment for COVID-19, only supportive care like oxygen, and mechanical ventilation is provided to patients in severe respiratory distress [2]. Two drugs, remdesevir and chloroquine, have shown some good results, but it is too soon to state their efficacy as there is limited data on the effectiveness of these drugs in COVID-19 patients. Researchers are also considering the passive immunization of critical patients with the convalescent sera, and it has shown some good results [8].

As we know, currently, there is limited knowledge available on the pathophysiology and treatment options for the COVID-19. And what little information is known is not well organized yet. In this review article, we aim to identify and highlight clinical features, pathological and radiological findings, and possible treatment options for COVID-19 patients and to organize the little information we have on this pathogen. 

## Review

Methods

Research Strategy

We conducted this research to analyze and review the clinical and pathological findings in COVID-19 affected patients. We used Pubmed and Google Scholar as our primary database research engines to find the relevant data. Keywords used were novel coronavirus, COVID-19, pathological findings, antivirals, treatment, management, and passive immunity. MeSH keyword coronavirus was also used.

*Inclusion/Exclusion criteria* 

We included papers from the past 5 years, they were mostly peer-reviewed, and full-text was available. All selected articles were in the English language, and no geographical boundaries were considered.

Results

We extracted 1683 (PubMed), and 56400 (Google scholar) researches using the keyword COVID-19. A combination of keywords coronavirus and pathological findings gave 1139 PubMed, and 22300 google scholar articles, and COVID-19 and treatment gave 385 PubMed articles. COVID-19 and management gave 167 published research papers. COVID19 and antivirals gave thirty-five PubMed articles. Lastly, COVID-19 and passive immunization yielded 287 results. Out of these results, a total of forty articles relevant to our topic were selected. After applying the inclusion-exclusion criteria and removing the duplications, we finally got twenty-nine papers that were reviewed. Among the selected papers, there were no clinical trials, most of the relevant papers were case reports which showed the clinical features and the effects of tried medical interventions. Some of them were simple recommendations and guidelines about how the patient should be managed. Three of these studies showed that a combination of lopinavir/Ritonavir combination was effective in decreasing viral load. Five studies argued that convalescent plasma showed promising results but most of these studies were not of high-quality evidence. Three other papers discussed the role of remdesivir, and five studies discussed the role of chloroquine in these patients which showed some positive effects however its safety profile is not clear. Table 1 shows the studies included.

**Table 1:** Some of the studies included in the review ARDS, acute respiratory distress syndrome [9-13]

Author, Year of study	Type of the study	Purpose of the study	Result/ Conclusion
Li T (2020)	Recommendation	To give comprehensive recommendations about Severe Acute Respiratory Syndrome Corona-virus 2 (COVID-19).	A combination of anti-virals Lopinavir/Ritonavir is useful in some cases and can be used as needed. When a patient develops ARDS, A protective ventilation strategy should be used, and when it is severe, the authors recommended extracorporeal membrane oxygenation.
Arabi et al. (2020)	Review article	This review summarized the knowledge about management of adult acute respiratory infections and community-acquired viral pneumonia patients that needed ICU.	More research and studies are needed to test different antiviral therapies both alone and in combination, to find the best option in patients who develop serious complications.
Lu H. (2020)	Review article	This study reviewed the literature to identify which drug can be a suitable option in COVID -19 infection	There are no specific antiviral drugs available and no vaccines for novel coronavirus. Based on previous experience with SARS and MERS, some antivirals are being used. However, their safety is yet to be established. The mainstay of treatment still is symptomatic support.
Lim et al. (2020)	Case Report	This case report reviewed the clinical findings and management in one of the patients of COVID-19, who was the first case of the tertiary spread of the virus out of China.	COVID-19 symptoms can be mild, and the patient may recover when the symptoms are mild but can transmit the disease. Lopinavir/Ritonavir combination reduced viral load and improved symptoms, so this combination can be used in high-risk patients. However, more clinical evidence is needed to establish its safety and efficacy.
Wang, Chao, et al. (2020)	Case series	Four patients with mild or severe COVID-19 improved after using lopinavir/ritonavir combination.	Three out of four patients improved clinically, especially their pneumonia-related symptoms, whereas others also showed significant improvement. More studies, however, are needed to establish their efficacy.

Discussion

Patient Presentation

COVID-19 may have produced intense effects worldwide due to rapid transmission and the fact that almost everyone is susceptible. Still, mostly the disease is mild, especially in children and immunocompetent adults [6]. A research paper written by Jin YH et al. divided different groups of exposed individuals under various categories of cases. These included the suspected cases (with two early clinical manifestations and an epidemiological risk), confirmed cases (with a positive RT-PCR result), close contacts (with contact history to a confirmed case of novel coronavirus without protection), clustered cases (one confirmed case + many cases showing symptoms in a small area within 14 days) and suspicious exposure [exposure to the contaminated environment without adequate protection [6]. This classification made it easy for a health professional to know what type of patient one is and how he should be managed. A patient may present with clinical features of the disease depending upon the severity of the disease. The different degrees are mild, severe, and critical [2]. Table 2 shows the clinical features of patients with varying degree of disease.

**Table 2:** Clinical features of patients with a varying degree of disease

Mild disease	Severe disease	Critical disease
Dry Cough	Fever	Respiratory failure
Fever	Tachypnea	Fever
Sour throat	Dyspnea	Decreases blood oxygen saturation
With or without nasal congestion		Septic shock
Generalized body aches		Multiple organ failure
Headache		
Malaise and fatigue		

Patients with mild disease presented with dry cough, fever, sore throat, with or without nasal congestion, generalized body ache, headache, and fatigue. In severe form, the symptoms were tachypnea and dyspnea. In critical cases, patients complained of respiratory failure (severe dyspnea, respiratory distress >30/min, tachypnea and hypoxia), fever, decreased blood oxygen saturation of less than or equal to 93%, lung filtrates, shock, and multiple organ dysfunctions or even failure [2,14]. Research also showed that in severe conditions, patients showed signs of weakened breath sound, moist rales, dull percussion, and a decrease in tactile speech tremor [6]. The disease causes not only respiratory symptoms but also gastrointestinal symptoms such as diarrhea [15]. Laboratory complete blood count tests may show normal or reduced white blood cell count or reduced lymphocyte count in the early stages of the disease onset [6,15]. Patients also show elevated C-reactive protein levels [16].

Researchers from China studied the effect of infection on pregnant women suffering from COVID-19 on the neonates. The study showed that the women that were confirmed cases during the prenatal stage had problems like premature rupture of membranes, intrauterine distress, abnormal amniotic fluid, umbilical cord, and position of the placenta. The neonates born also presented with shortness of breath, fever, tachycardia, vomiting, inability to take proper feed, diarrhea, and bloating [5]. The complications are mostly seen in patients with an underlying chronic illness or are old. Complications included acute respiratory distress syndrome (ARDS), RNAemia, acute cardiac injury, and secondary infection. The patients with complications were admitted to the intensive care unit (ICU) [15-16].

These clinical features indicate that the absence of these symptoms in an individual does not rule out the diagnosis of COVID-19. If a person has a history of exposure to the virus regardless of the symptoms that he shows, he should be kept under medical observation, isolated from others, and examined thoroughly [16]. The diagnosis should be made on history, examination, symptoms, and certain investigations that are necessary and gold standard in virus detection (RT-PCR, CT scan, POCT of IgG and IgM, ELISA, and blood culture) [4].

According to the reviewed data, patients had similar presenting features and progression of the disease with some minor differences according to the individual immune status of the subject and how they were treated in their health settings. The majority recovers without complications;deaths involve the high-risk group like the geriatric age group or persons with co-morbidities. The consistent findings were fever, dry cough, sore throat, myalgia, fatigue, and shortness of breath. The distinct findings in different cases were tactile speech tremor, GI symptoms, hemoptysis, ARDS, and septic shock [2,6,15,17-18].

Pathological and Radiological Findings In COVID-19 Patients

**Figure 1:**
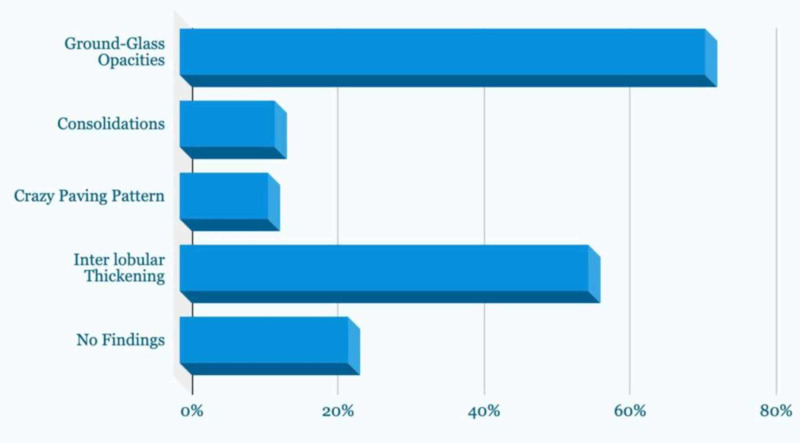
Pathological findings of patients as per study done by Xu X et al.

Radiological findings: A case review series by Lin X et al. showed that both patients had ground-glass opacities on CT scans, which later progressed to patchy consolidation of lungs. The lesions were mostly distributed along the subpleural regions in both lungs. No pleural effusion or lymphadenopathy was observed in subjects included in this study [19]. Another case report by Shi F et al. showed that in this subject initially, ground-glass opacities were seen on CT scan, which later on progressed to consolidation. On the 18th day, the patient had increased consolidation, and the patient showed diffuse haziness in both lungs. Other findings were bat wing appearance, crazy paving pattern, and air-bronchogram sign. The lesions involved the peripheral regions of both the lungs [17]. Another study conducted by Xu X et al., which included 90 patients out of which 39 were males and 51 females. The median age of the patients was 50 years. More than 50% of the patients had bilateral multifocal lesions in their lungs distributed along the periphery. Pneumonia in these patients presented with ground-glass opacities in 72%, consolidations in 13%, crazy paving pattern in 12%, interlobular thickening in 56% and adjacent pleural thickening in 56% of patients. Twenty-three percent of the patients did not show any of these findings [16]. Figure 1 summarizes the findings of this research. 

**Figure 2:**
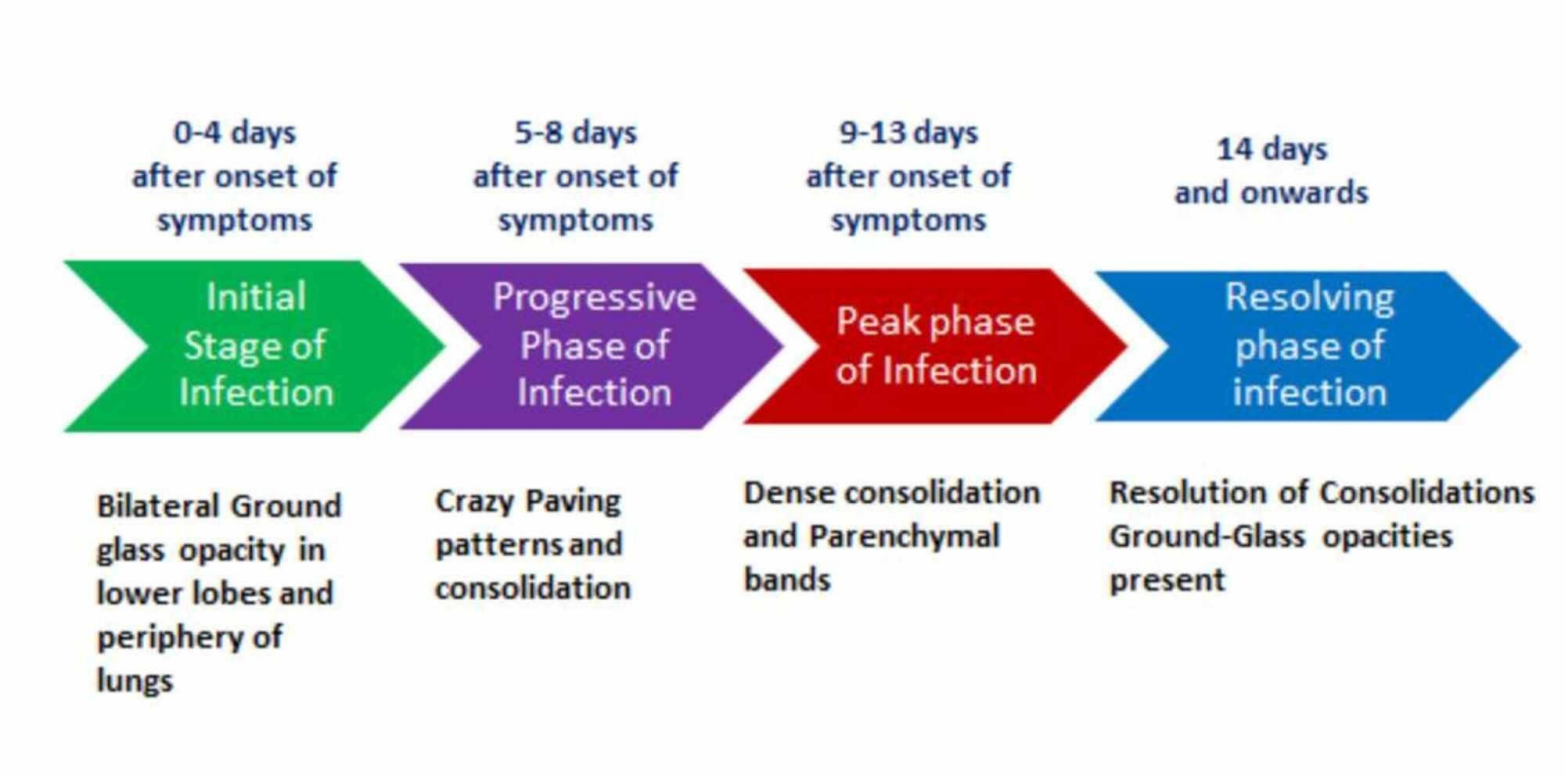
Progression of CT findings in COVID-19 patients CT, computed tomography

Uncommon findings were pleural effusion, thoracic lymphadenopathy, pulmonary emphysema, and pericardial effusion. CT scan of 52 patients was redone after six days, 19% of them showed no changes in CT scan while 4% had resolution of the disease, and 73% showed progression. 6% of the patients who didn’t have any characteristic findings on a previous CT scan developed bilateral ground-glass opacities [16]. Another study carried out by Albarello F, et al. based on two cases showed somewhat uncommon findings. The chest X-ray of the two patients showed interstitial involvement, lung opacities, pleural effusion and calcification, hilar enlargement, and cardiac silhouette. Their CT findings were typical ones like ground-glass opacities with consolidation. Some uncommon findings like pleural effusion, calcifications, mediastinal lymphadenopathy, and pericardial effusions were also seen [20]. A study carried out by Pan F et al. showed the different phases of recovery in patients, excluding the patients who developed ARDS and severe pneumonia. The study included a total of 21 patients with confirmed COVID-19 diagnosis. The study showed there are four stages in the disease progression based on CT findings. Early-stage (0-4 days after the onset of initial symptoms) of the disease showed unilateral or bilateral ground-glass opacities, mostly in the lower lobes of lungs. Progressive stage (5-8 days after the onset of initial symptoms) in this stage ground-glass opacities progressed to crazy paving patterns and consolidation. Peak stage (9-13 days after the onset of initial symptoms) consolidations became denser and more prevalent, and there were residual parenchymal bands. Absorption Stage (14 or more days after the onset of initial symptoms) consolidation gradually absorbed, there was no crazy paving pattern observed, but extensive ground-glass opacities were still observed [21]. Figure 2 shows the CT findings in COVID-19 patients.

Based on the studies reviewed, the findings on the CT scan of COVID-19 patients are ground-glass opacities, consolidation, crazy paving pattern, and air bronchogram sign. These findings are consistent with the majority of the patients, and these are the characteristic findings of COVID-19. In some patients, mediastinal lymphadenopathy, pleural effusion, pulmonary emphysema, and pericardial effusion are seen and are mostly in older patients and the high-risk groups. CT scan findings of COVID-19 patients are also helpful in determining the severity of disease and the stage of the disease. Also, in the areas where they have limited testing services, CT scan findings can be used as a diagnostic tool for COVID-19. There is a specific pattern in which these findings present at the initial stages of the infection, ground-glass opacities are frequently bilateral and present in the lower lobes and the periphery. They may then progress to develop consolidation and crazy paving patterns. Consolidation will start becoming denser, and parenchymal bands will appear, followed by a peak. In most cases, consolidation starts resolving, and the CT scan becomes normal again within two weeks. Therefore, a CT scan can be used to monitor the progression of the disease.

Pathological Findings: A study conducted by Luo W, et al. based on the autopsy findings of the patient who died of COVID-19. On gross examination of the lungs, the surface appears diffusely congested, hemorrhagic necrosis was evident and was more marked in the outer regions of the lungs. Mucinous and hemorrhagic exudates were present in the bronchi. On histopathology, extensive pulmonary interstitial fibrosis with hyaline degeneration was evident. There were signs of a hemorrhagic infarct. Small vessels showed severe congestion, thickening of walls and occlusion or stenosis of the blood vessel; lumen and microthrombi were present in the vessels. Focal infiltration of lymphocytes, plasma cells, macrophages was marked. There was marked necrosis of bronchial walls. Mainly type 2 pneumocytes were affected, and various metaplastic changes of epithelium were observed. Multinucleated giant cells and intracytoplasmic viral inclusions were also observed [22]. Another study conducted by Xu Z et al. based on the biopsy samples taken from the deceased COVID-19 patient showed diffuse alveolar damage bilaterally with cellular fibromyxoid exudates. There was the formation of the hyaline membrane and interstitial infiltration of inflammatory cells. Pneumocytes were atypically enlarged, having large nuclei, granular cytoplasm, and prominent nucleoli showing the viral cytopathic change [18]. Another study conducted by Zhang H et al. based on the biopsy findings of deceased COVID-19 patients showed diffuse alveolar damage and type 2 pneumocyte hyperplasia. Fibrinous exudates were seen in the alveoli, and interstitial fibrosis was evident. Interstitial infiltration of inflammatory cells was also observed [23]. Based on the studies reviewed, on gross examination, lungs were markedly congested, and hemorrhagic necrosis was seen, and several exudates were present in bronchi. On the other hand, characteristic histopathological findings were severe pulmonary interstitial fibrosis, hemorrhagic infarction. Small vessels were mainly affected, and they showed signs of congestion and were occluded by microthrombi. Type 2 pneumocytes were the primary cells that affected their nuclei were atypically enlarged, and nucleoli were prominent. Metaplastic change in the epithelium was seen. Infiltration of inflammatory cells i-e lymphocytes, plasma cells, macrophages were marked, showing a severe inflammatory reaction. Multinucleated giant cells were also seen, and some studies also suggested the presence of intracytoplasmic viral inclusions.

Prevention and Treatmentof COVID-19

Several options are in use and proposed to prevent COVID-19 infection. Some are simple preventive measures suggested to the general public, like frequent hand washing, social distancing, self-isolating if feeling sick. These measures proved to be effective in flattening the curve of COVID-19 infection and keeping the total cases within the healthcare system’s capabilities. It is proposed that any person who is exposed to the confirmed case of COVID-19 or is suspected of having these symptoms should practice self-isolation. This will prevent passing the virus to other healthy and vulnerable individuals; however, once they develop symptoms like fever or cough, it is advisable to consult a physician to get tested.

For mild symptoms the advice is different in different geographical regions, some advise the COVID-19-positive individuals with mild symptoms to quarantine at home, practice self-isolation, keep a check on their symptoms along with symptomatic measures like antipyretic. Whereas in some regions where the healthcare system is not overwhelmed, they are being held in quarantine under supervision until they test negative to avoid it’s spread [9]. For moderate to severe symptoms, however, the patients are shifted to the hospital and are managed there. Some of the patients do progress to severe form of the disease or ARDS and multi-organ failure and then need ICU care and other supportive measures.

Several factors cause the progression of the disease from mild symptoms to severe and poor prognosis of the disease. One of these factors is old age, where comorbidities and polypharmacy make them vulnerable and worsen their symptoms. Other factors include co-morbidities and status of the existing disease like increased d-dimer levels and high sepsis-related organ assessment score (SOFA score) for sepsis.

**Figure 3:**
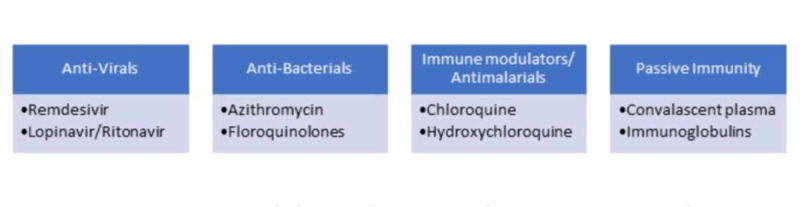
Treatment options for COVID-19

Although there is no definitive treatment available when the patient does develop symptoms, he is managed using one of the proposed treatment options along with other supportive options. These proposed treatment options are based on the previous epidemics because of other coronaviruses like SARS and MERS CoV. Figure 3 shows some of those options.

Antivirals: One of the mainstays of treatment remains antivirals in any viral infection. Among antivirals are the neuraminidase inhibitors which worked in SARS and MERS, Oseltamivir is one of those and is a widely used anti-viral. Arabi et al. found zanamivir and Panamivir to be as effective as oseltamivir in patients with influenza and H1N1 infections [10]. However, these anti-virals are not that effective in COVID-19 patients. Although there is not enough evidence to support their effectiveness in this latest outbreak, the combination of lopinavir/ritonavir is found to be effective in some patients. A case report by Lim et al. showed that the combination of lopinavir and ritonavir significantly reduced the viral load in a 54-year-old patient [12]. Another case series by Wang Z et al. showed the results of this combination in four patients where severe pneumonia and respiratory symptoms significantly improved, and one patient tested negative and was discharged later [24]. Remdesivir, another broad-spectrum antiviral, is being used in these patients to find potential treatment and is among the ones that are relatively effective in COVID-19 [11]. Elfiky showed in a study that Sofosbuvir, Remdesivir, and Ribavirin showed that they could help manage and treat this virus [25].

Antibacterials: Superimposed bacterial infections are common in patients with viral pneumonia, and use of antibacterial medications alongside as prophylaxis in moderate to severe infections or wherever there is suspected superimposed infection. It is, however, recommended to avoid a combination of broad-spectrum antibiotics or their blind administration [6, 9]. The antibiotic treatment recommended for community-acquired pneumonia, like Azithromycin, fluoroquinolones can be administered to cover common bacterial pathogens. 

Immune modulator drugs: Chloroquine and hydroxychloroquine, the immune modulator drugs used as antimalarials, and as a DMARD in Rheumatoid Arthritis and also in autoimmune diseases like Lupus were found effective in SARS infection and so were tried in COVID-19 patients as well. They also act as a broad-spectrum antiviral drug. Several clinical trials are in progress throughout the world to assess its efficacy and safety in these patients. An in-vitro study testing the efficacy of broad-spectrum anti-virals like remdesivir, favipiravir, ribavirin penciclovir, and chloroquine showed that chloroquine and remdesivir were effective against COVID-19 virus in-vitro [13]. Another study involving 100 patients by Gao et al. also showed similar results; it showed an increased efficacy of chloroquine in preventing symptom exacerbation, preventing progression to severe pneumonia, and improving radiological findings without any serious side effects [26]. Chloroquine has both anti-viral and immune-modulating properties, which increase its effectiveness in stopping the viral replication in vivo [13,26]. Chloroquine, however, is associated with some uncommon but serious side-effects like QT interval prolongation, which may be severely harmful in cardiac patients. Therefore, more clinical trials are needed to assess its safety profile in this group of patients.

Convalescent plasma and passive immunization: Convalescent plasma has already been in use for some diseases; it was also effective in MERS-CoV infection. Its safety and efficacy were tested by Arabi et al. and showed that it could be used as a potential treatment option in COVID-19 [27]. The WHO recently recommended its use as passive immunization in all serious patients because of the unavailability of active immunization in the form of vaccines [27].The plasma of recovered patients was used in different patients and had shown positive effects. A case review series by Shen et al. discussed five critically ill patients having COVID-19 with ARDS who were given antibody-rich convalescent plasma. They showed significant improvement in clinical status, their SOFA score improved, temperature improved, and they also were weaned off from a ventilator [28]. The results, however, cannot be considered as significantly reliable because of the small sample size. Another case study showed significant improvement in symptoms and a decrease in viremia and viral load in seven out of 10 patients without any serious side effects [29]. Despite the positive results and considering that most of the studies had weak evidence, it can help manage patients. Still, more studies are needed to assess its potential serious side-effects like transfusion-related acute lung injury (TRALI).

Supportive treatment: Supportive treatment like oxygen therapy via mask is used if patients develop shortness of breath and hypoxemia. In the case of resistant hypoxemia, Extracorporeal membrane oxygenation (ECMO) is used. Patients with severe respiratory Distress may need ventilator support. Corticosteroids may be used as a supportive therapy, but its use and efficacy are controversial as they may make patients more prone to infections [9].

Limitations

Most of the clinical trials are currently ongoing. Most of them involved fewer patients, which makes it difficult to make valid conclusions. Only the papers that were published in the English language were selected for our review. Most of the interventions are under trial; therefore, not much data was available about their safety in this population, and thus conclusions were hard to draw and are not valid. A detailed quality appraisal of the papers was not done.

## Conclusions

The research was oriented to identify clinical features, pathological and radiological findings, and possible treatment options of COVID-19. The majority of the patients experience fever, cough, shortness of breath, and myalgia whereas some may develop pneumonia and ARDS. Frequently seen radiological and pathological findings are bilateral ground-glass opacities in peripheries of lungs, consolidations, crazy paving pattern, air bronchogram sign, diffuse alveolar damage, fibrinous exudates in bronchi, formation of hyaline membrane, inflammatory cells infiltration, and metaplastic change in the alveolar epithelium. There is no definitive treatment, but the role of antivirals like remdesivir and lopinavir, azithromycin, hydroxychloroquine, and passive immunization like convalascent plasma is being researched. Currently, the focus is on the prevention of disease by practicing social distancing until a vaccine is developed. There is a great need for more work on vaccine development and educating the medical community and the public. Strict protocols are needed in the future to prevent such crises.
